# Syndemic mapping of HIV and other STIs in KwaZulu-Natal: a Bayesian spatio-temporal modeling approach using latent constructs

**DOI:** 10.3389/fpubh.2025.1683985

**Published:** 2025-10-14

**Authors:** Exaverio Chireshe, Retius Chifurira, Jesca Mercy Batidzirai, Knowledge Chinhamu, Ayesha B. M. Kharsany

**Affiliations:** ^1^Statistics, School of Agriculture and Science, College of Agriculture, Engineering and Science, University of KwaZulu-Natal, Westville Campus, Durban, South Africa; ^2^Statistics, School of Agriculture and Science, College of Agriculture, Engineering and Science, University of KwaZulu-Natal, Pietermaritzburg Campus, Pietermaritzburg, South Africa; ^3^Centre for the AIDS Programme of Research in South Africa (CAPRISA), University of KwaZulu-Natal, Durban, South Africa; ^4^School of Laboratory Medicine and Medical Sciences, Nelson R Mandela School of Medicine, University of KwaZulu-Natal, Durban, South Africa

**Keywords:** syndemics, HIV, STIs, spatio-temporal, bayesian modeling, latent variable

## Abstract

Syndemics involving Human immunodeficiency virus (HIV) and other sexually transmitted infections (STIs) remain a major public health challenge in sub-Saharan Africa, and understanding their spatial and temporal dynamics is critical for effective interventions. Using data from two consecutive, population-based cross-sectional surveys conducted in 2014 and 2015 under the HIV Incidence Provincial Surveillance System (HIPSS) in KwaZulu-Natal, South Africa, we applied a Bayesian spatio-temporal framework grounded in latent variable modeling to quantify and map the syndemic burden of HIV and other STIs. A confirmatory factor analysis constructed a continuous latent syndemic score from four binary indicators (HIV diagnosis, HIV testing, STI diagnosis, and STI symptoms), which was modeled using Bayesian hierarchical spatial methods via Integrated Nested Laplace Approximation (INLA), incorporating spatial random effects through the Stochastic Partial Differential Equation (SPDE) approach and temporal effects via a first-order random walk. Local spatial autocorrelation, assessed using Local Moran's *I* and Getis-Ord Gi^*^ statistics, revealed consistent hotspots and coldspots. Syndemic burden of HIV and other STIs was higher among younger adults (20–49 years), women, individuals with incomplete secondary education, those engaging in sexual risk behaviors or reporting forced sexual debut, and those facing socioeconomic vulnerabilities such as food insecurity. Access to healthcare and treatment for depression were also positively associated, likely reflecting increased detection. Local Moran's *I* identified 11 significant clusters (three hotspots, eight coldspots), and Getis-Ord Gi^*^ identified 32 (17 hotspots, 15 coldspots), with hotspot patterns persisting across both years, indicating temporal stability. These findings highlight the utility of Bayesian latent variable and spatio-temporal modeling in integrating multiple co-occurring health conditions into a single spatial framework, providing actionable evidence to support geographically targeted, multi-sectoral interventions that address structural and behavioral drivers of co-epidemics in resource-limited settings.

## 1 Introduction

The intersecting epidemics of Human immunodeficiency virus (HIV) and other sexually transmitted infections (STIs) represent a persistent and synergistic public health challenge in sub-Saharan Africa, particularly in South Africa, which bears one of the highest HIV burdens globally (1). Within the country, the province of KwaZulu-Natal (KZN) remains at the epicenter of this crisis, with sustained high HIV prevalence (2, 3).

These infections often do not occur in isolation; rather, they co-occur and interact synergistically within structurally disadvantaged populations. This clustering and mutual enhancement of diseases, especially under the influence of social, economic, and environmental stressors, is known as a syndemic (4, 5). The syndemic framework emphasizes the interaction of epidemics driven by common social determinants such as poverty, gender inequality, limited healthcare access, and food insecurity (6, 7). Despite its conceptual relevance, empirical applications of syndemic theory, especially those incorporating spatial and temporal dimensions, remain limited in resource-constrained, high-prevalence settings like KZN.

Significant progress has been made in mapping the individual epidemiology of HIV and other STIs (8, 9). However, most studies examine these diseases in isolation, often using traditional univariate approaches that overlook spatial correlation, temporal trends, or shared structural drivers. Studies such as (9, 10) highlight spatial heterogeneity in HIV and syphilis, respectively, but rarely account for joint clustering or interdependence between diseases. Even where repeated population-based data like the HIV Incidence Provincial Surveillance System (HIPSS) exist (11), comprehensive syndemic modeling remains underdeveloped.

Recent methodological advances in spatial epidemiology offer promising opportunities to bridge these gaps. Latent variable models, particularly within a structural equation modeling (SEM) framework, can combine multiple observed indicators (e.g., HIV testing, STI symptoms) into a single continuous latent construct that reflects the underlying disease burden more holistically (12, 13). When integrated within a Bayesian hierarchical framework, these models accommodate spatial dependency and uncertainty (14, 15). In addition, the stochastic partial differential equation (SPDE) approach enables fine-scale spatial modeling with continuous surfaces and quantified uncertainty (16).

Despite these methodological innovations, few studies have integrated latent variable modeling with spatial and temporal analyses into a unified framework. Although the utility of spatial modeling for understanding HIV epidemics has been well demonstrated (17), these studies did not employ latent constructs or Bayesian spatio-temporal models to capture syndemic interactions.

This study addresses these critical gaps by applying a Bayesian spatio-temporal structural equation modeling framework to jointly model HIV and STI syndemics in KwaZulu-Natal. Specifically, we construct a latent *Syndemic Score* through confirmatory factor analysis and model it using spatially and temporally structured random effects via SPDE and random walk priors. Furthermore, we identify syndemic hotspots using Local Moran's *I* and Getis-Ord Gi^*^ statistics, and explore associations with a range of demographic, behavioral, and structural covariates.

By uniting latent variable theory with advanced Bayesian spatio-temporal techniques, this study contributes both to methodological innovation and practical relevance. It models the syndemic burden not merely as a statistical construct but as a meaningful, spatially and temporally dynamic phenomenon. The resulting maps and insights provide an evidence base for designing targeted, multi-sectoral health interventions in resource-limited settings like KZN.

## 2 Materials and methods

### 2.1 Sources of data and study population

This study utilizes secondary data derived from two successive population-based cross-sectional surveys conducted as part of the HIV Incidence Provincial Surveillance System (HIPSS). HIPSS was a comprehensive surveillance program established to track trends in HIV prevalence and incidence within KwaZulu-Natal, South Africa. The first wave of data collection took place from 11 June 2014 to 18 June 2015, followed by a second round from 8 July 2015 to 7 June 2016. Both surveys were implemented in Vulindlela, a rural community, and Greater Edendale, a peri-urban setting, both located in uMgungundlovu District.

To achieve a representative sample, HIPSS employed a multistage probability sampling framework. Of the 600 available enumeration areas (EAs), 591 containing at least 50 households qualified. A random sample of 221 EAs was selected for the 2014 round and 203 for the 2015 round. Households within these areas were systematically chosen, and from each, one eligible individual was randomly selected upon obtaining written informed consent. GPS coordinates were recorded for each sampled household to facilitate geospatial analysis and reduce potential selection bias.

Data integrity was upheld through rigorous quality assurance protocols. Daily monitoring occurred during the initial month of fieldwork, followed by monthly audits for the next 6 months and quarterly reviews thereafter. Field operations were managed using the Mobenzi Researcher system (Durban, South Africa), which enabled real-time tracking of data collection, protocol adherence, and data accuracy. Built-in validation checks flagged inconsistencies promptly for correction. The dataset includes laboratory-confirmed HIV test outcomes from blood samples, further strengthening its epidemiological robustness. All records underwent central processing with thorough quality control and completeness checks.

A total of 20,048 individuals aged 15–49 years participated in both survey waves, 9,812 in 2014 (6,265 women and 3,547 men) and 10,236 in 2015 (6,431 women and 3,805 men). Several variables had missing data, ranging from negligible (< 1% for *MainIncome, MealCutsYear, MealSkipYear, RunOutMoneyYear, AccessedHealthCare*) to moderate (*STISymptoms*, 7%; *ForcedFirstSex*, 14%). To manage incomplete data, multiple imputation using the mice package in R was carried out, with appropriate models applied for categorical and continuous variables. All participants were retained for inclusion in the final analyses. Complete case analysis was deemed inappropriate as it would have reduced the sample, potentially introducing severe bias. Although some degree of bias may be unavoidable, the strong sampling design and careful handling of missing data through imputation enhance the reliability and generalisability of the findings.

The decision to concentrate on individuals aged 15–49 years is informed by both public health priorities and international reporting conventions. This demographic represents the core of the sexually active and economically productive population, who are disproportionately affected by HIV and other STIs. Furthermore, this age range is consistent with national HIV surveillance protocols and global monitoring frameworks such as those established by UNAIDS. As a result, the findings are directly comparable to broader epidemiological data and can effectively inform targeted interventions in high-burden areas like KwaZulu-Natal.

### 2.2 Syndemic construct and study variables

In line with the syndemic theory framework, the co-occurrence and mutual reinforcement of HIV and other STIs were conceptualized as a single latent construct. This latent syndemic construct was derived from four binary observed indicators: HIV status, HIV testing history, STI diagnosis, and self-reported STI symptoms. All indicators were standardized prior to analysis.

To quantify the latent syndemic burden, Confirmatory Factor Analysis (CFA) was conducted using the lavaan package in R. The Diagonally Weighted Least Squares (DWLS) estimator, appropriate for categorical data, was employed to ensure robust parameter estimation. Model fit was evaluated using conventional thresholds: a Comparative Fit Index (CFI) above 0.95, a Root Mean Square Error of Approximation (RMSEA) below 0.06, and a Standardized Root Mean Square Residual (SRMR) below 0.08 were considered indicative of good fit.

All categorical covariates were converted into factor variables, while binary variables were recoded to reflect binary responses as 1 (Yes) and 0 (No). The covariates considered in the analysis spanned several domains. Demographic variables included age group, gender, marital status, education level, and primary source of income. Behavioral variables encompassed alcohol use, sexual initiation, and number of sexual partners. Structural variables captured indicators of socioeconomic vulnerability and healthcare access, including food insecurity (measured by meal skipping, meal cutting, and running out of money), forced first sex, migration status (away from home), and level of community attachment. Additionally, a binary variable was included to capture mental health service utilization, specifically treatment for depression.

Individual-level latent syndemic scores were subsequently extracted using the lavPredict() function and served as the primary outcome variable in subsequent modeling. These scores were then analyzed within a Bayesian hierarchical spatio-temporal framework, allowing for the investigation of their associations with sociodemographic, behavioral, and structural covariates, as well as their geographic and temporal distribution across the study region. This approach enabled the identification of syndemic burden hotspots and temporal trends in KwaZulu-Natal.

The extracted syndemic scores were summarized using means, standard deviations, and quantiles. Their distributional properties were assessed using histograms, density plots, and skewness and kurtosis statistics.

### 2.3 Statistical analysis

The analytical strategy focused on modeling the latent syndemic score using a Bayesian hierarchical framework to assess both its spatial distribution and temporal dynamics. The continuous syndemic score, derived from confirmatory factor analysis, served as the primary outcome. To account for geographic and temporal correlation, we employed the Integrated Nested Laplace Approximation (INLA) approach (18), incorporating all individual- and household-level covariates as fixed effects. Individual survey weights (weight_individual) were applied in the INLA model to account for the complex survey design, including differential probabilities of selection at the cluster, household, and individual levels, as well as non-response adjustments. Incorporating these weights ensures that the estimates are representative of the target population and enhances their generalisability.

The spatial component was modeled using the Stochastic Partial Differential Equation (SPDE) method, which approximates a Gaussian random field as a Gaussian Markov random field, allowing continuous spatial modeling over the study region (16). Longitude and latitude coordinates for each observation were used to construct a two-dimensional spatial mesh, with spatial effects estimated directly from these point-level locations rather than from spatial polygons or area-level summaries. To further explore local geographic patterns, Local Indicators of Spatial Association (LISA) and Getis-Ord Gi^*^ statistics were computed to identify significant clustering and hotspot areas.

Enumeration Areas (EAs), defined by Statistics South Africa as the smallest census-based geographic units used for household enumeration (typically encompassing 50–150 households), served as the primary sampling units in the survey design and as the basis for spatial clustering in the analysis. In the modeling framework, EA-level information was incorporated via an EA-by-year random effect to capture unstructured spatio-temporal heterogeneity. Temporal correlation was explicitly modeled as a first-order random walk over survey years. This combined strategy allowed us to exploit the fine-scale information from household coordinates, while ensuring that residual variation at the EA and EA-by-year levels was appropriately accounted for.

Temporality was modeled explicitly by including a first-order random walk (RW1) prior on survey year in the INLA framework. This allows consecutive years to be correlated, capturing smooth temporal evolution of the latent syndemic burden. The RW1 prior assumes that each year's effect depends on the previous year with Gaussian-distributed differences, while being constrained to sum to zero for identifiability. This specification enabled us to quantify deviations in syndemic burden across survey years while accounting for residual space–time interaction.

#### 2.3.1 Prior specification and model comparison

We fitted two versions of the Bayesian spatio-temporal syndemic model that differed in their prior specifications. The first model (Model 1) relied on the default priors in INLA: fixed effects were assigned vague Gaussian priors (mean 0, precision 0.001), while variance parameters for temporal, spatial, and interaction components followed vague Gamma priors (shape 1, rate 0.00005). The second model (Model 2) employed explicit penalized complexity (PC) priors, while still using vague Gaussian priors for fixed effects. For the temporal component, we specified a random walk of order 1 (RW1) with a PC prior on its precision [prec ~ PC.prec(1, 0.01)], which implies a 1% probability that the standard deviation of the temporal effect exceeds 1. The space–time interaction, modeled as an independent and identically distributed (IID) random effect, was also assigned a PC prior on its precision with the same parameterization. The spatial field was represented through the SPDE approach, with PC priors jointly placed on the range (to avoid unrealistically short correlation distances) and on the marginal precision. PC priors were chosen because they are weakly informative and penalize deviation from simpler base models (e.g., no spatial or temporal structure), thereby reducing the risk of overfitting while retaining interpretability. Comparing the default-prior model with the explicit-PC-prior model allowed us to assess the robustness of our findings to alternative prior assumptions.

#### 2.3.2 Latent variable structural equation model (measurement model)

To quantify the latent burden of HIV and STIs, a confirmatory factor analysis (CFA) was used to define a latent syndemic construct based on four observed binary indicators. This measurement model allows multiple correlated outcomes to be represented by a single underlying factor (19, 20) as expressed in Equation 1:


(1) $$\begin{array}{r} Syndemic={\text{λ}}_{1}\cdot HIV_{tested}+{\text{λ}}_{2}\cdot HIV_{status}+{\text{λ}}_{3}\cdot STIs_{Symptoms}\\ +{\text{λ}}_{4}\cdot STIs_{diagnosed}+\epsilon . \end{array}$$

The CFA model for each individual *i* and indicator *j* is given by Equation 2:


(2) $$\begin{array}{l} y_{ij}={\text{λ}}_{j}\eta_{i}+\epsilon_{ij},\;\epsilon_{ij}\sim N\left(0,{\sigma^{2}}_{j}\right), \end{array}$$

Or in matrix form as in Equation 3:


(3) $$\begin{array}{l} y_{i}=\text{Λ}\eta_{i}+\epsilon_{i},\epsilon_{i}\;\sim N\left(0,\text{Ψ}\right), \end{array}$$

where *y*_*i*_ is the observed vector of outcomes for individual *i*, Λ is the loading matrix, η_*i*_ is the individual latent score, and Ψ is the covariance of residuals.

#### 2.3.3 Structural model (regression on covariates)

The syndemic scores were regressed on a set of socio-demographic, behavioral, and spatial-temporal covariates using a Bayesian hierarchical framework. The structural equation is given in Equation 4:


(4) $$\begin{array}{l} y_{i}=\beta_{0}+\overset{K}{\underset{k=1}{\sum }}\beta_{k}X_{ik}+f_{temporal}\left(t_{i}\right)+f_{spatial}\left(s_{i}\right)+f_{s-t}\left(s_{i},t_{i}\right)+\epsilon_{i}, \end{array}$$

where *f*_*temporal*_, *f*_*spatial*_, and *f*_*s*−*t*_ represent random effects capturing temporal trend, spatial structure, and space-time interaction, respectively. This approach captures latent structure while accounting for spatial autocorrelation and temporal dynamics (21, 22).

The temporal effect was modeled as Equation 5:


(5) $$f_{year}\left(t\right)-f_{year}\left(t-1\right)=\epsilon_{t,\;}\epsilon_{t}\sim N(0{,}^{-1}tme),$$

where _*time*_ is the precision for the temporal effect.

The spatial effect was specified using the stochastic partial differential equation (SPDE) method (Lindgren et al., 2011 ) shown in Equation 6:


(6) $$\begin{array}{l} {\left(\kappa^{2}-\text{Δ}\right)}^{\frac{\alpha }{2}}=\;W\left(s\right), \end{array}$$

where *W*(*s*) is the spatial white noise, Δ is the Laplacian operator, κ is the spatial scale parameter related to spatial range *r* and α defines Matérn covariance smoothness.

The spatial range parameter is linked to the scale κ as expressed in Equation 7:


(7) $$\begin{array}{l} r=\frac{\sqrt{8}}{\kappa }. \end{array}$$

The SPDE approach implies a Matérn covariance structure given in Equation 8:


(8) $$\begin{array}{r} Cov\left(f\left(s_{i}\right),f\left(s_{j}\right)\right)=\sigma^{2}\cdot \frac{1}{2^{\nu -1}\Gamma \left(\nu \right)}{\left(\kappa ∥s_{i}-s_{j}∥\right)}_{\nu }^{\nu }\\ \left(\kappa ∥s_{i}-s_{j}∥\right), \end{array}$$

where _ν_ is the modified Bessel function and ν controls smoothness.

#### 2.3.4 Likelihood specification

Assuming Gaussian outcomes, the likelihood function for the *i*-th observation is given in Equation 9:


(9) $$\begin{array}{l} L\left(y_{i}|\text{θ}\right)=\frac{1}{\sqrt{2\pi \sigma^{2}}}\exp\left(-\frac{1}{2\sigma^{2}}{\left[y_{i}-\;\mu_{i}\right]}^{2}\right), \end{array}$$

(10) $$\text{where }\mu_{i}=X_{i}\beta +f_{spatial}\left(s_{i}\right)+f_{temporal}\left(t_{i}\right)+f_{s-t}\left(s_{i},t_{i}\right),$$

(11) $$\begin{array}{l} \text{and }\theta =\left\{\beta ,f_{spatial}\left(s_{i}\right),f_{temporal}\left(t_{i}\right),f_{s-t}\left(s_{i},t_{i}\right),\sigma^{2}\right\}. \end{array}$$

Equation 10 explicitly defines the structure of the linear predictor, while Equation 11 lists all parameters for which the likelihood in Equation 9 is conditioned.

Assuming conditional independence, the full likelihood is given by Equation 12:


(12) $$\begin{array}{l} L\left(\text{y}|\text{θ}\right)=\overset{n}{\underset{i=1}{\prod }}\left[\frac{1}{\sqrt{2\pi \sigma^{2}}}\exp\left(-\frac{1}{2\sigma^{2}}{\left[y_{i}-\;\mu_{i}\right]}^{2}\right)\right]. \end{array}$$

The corresponding log-likelihood is expressed in Equation 13:


(13) $$\begin{array}{l} log\;L\left(y|\theta \right)=-\frac{n}{2}\;log\;2\pi \sigma^{2}-\frac{1}{2\sigma^{2}}\overset{n}{\underset{i=1}{\sum }}{\left(y_{i}-\;\mu_{i}\right)}^{2} \end{array}$$

#### 2.3.5 Bayesian inference and posterior distribution

Following Bayes' theorem, the joint posterior distribution is proportional to the product of the likelihood and the priors (12):


$$\begin{array}{l} p\left(\;\theta |y\right)\propto \;L\left(y|\theta \right)\cdot p\left(\theta \right) \end{array}$$

#### 2.3.6 Marginal posterior and predictive distributions

The marginal posterior of a parameter θ_*j*_ is shown in Equation 14:


(14) $$\begin{array}{l} p\left(\theta_{j}|y\right)=\int p\left(\theta |y\right)d\theta_{-j}, \end{array}$$

where θ is the full vector of all parameters, θ_−*j*_ are all parameters except θ_*j*_ and *y* are the observed indicators.

The posterior predictive distribution for a future observation ${y^{rep}}_{i}$ is given as Equation 15:


(15) $$\begin{array}{l} p\left({y^{rep}}_{i}|y\right)=\int p\left({y^{rep}}_{i}|\theta \right)\cdot p\left(\theta |y\right)d\theta , \end{array}$$

For Gaussian likelihoods:


$$\begin{array}{l} {y^{rep}}_{i}|\theta \sim N\left(\mu_{i},\sigma^{2}\right), \end{array}$$

The posterior predictive distribution is given as Equation 16:


(16) $$\begin{array}{l} p\left({y^{rep}}_{i}|\text{y}\right)=\int N\left({y^{rep}}_{i}|\mu_{i},\sigma^{2}\right)\cdot p\left(\theta |y\right)d\theta . \end{array}$$

### 2.4 Model diagnostics

To ensure the validity and reliability of the fitted Bayesian spatio-temporal structural equation model, a comprehensive set of model diagnostics was conducted. Model fit was assessed using the Deviance Information Criterion (DIC) and the Watanabe-Akaike Information Criterion (WAIC), both of which balance model complexity and goodness of fit. Conditional Predictive Ordinate (CPO) and Probability Integral Transform (PIT) values were computed to assess model predictive performance and calibration. A histogram of PIT values that approximates a uniform distribution suggests good model calibration. Additionally, the absence of extreme outliers in the log(CPO) plots further supports satisfactory predictive adequacy. Convergence and precision of the parameter estimates were examined by comparing posterior means and modes, and by inspecting Kullback–Leibler divergence (KLD) values. KLD values near zero indicate stable posterior distributions. Furthermore, posterior density plots of the fixed effects were visually inspected to confirm unimodality and smoothness, providing additional support for convergence. Lastly, hyperparameters such as the spatial range, standard deviation, and precision terms were examined to evaluate the contribution and stability of the random effects components. All diagnostic procedures were conducted in R using the INLA package and relevant spatial libraries.

### 2.5 Software and implementation

All statistical and spatial analyses were implemented in R (version 4.5.1) using the R-INLA package for Bayesian inference. Confirmatory factor analysis was conducted using the *lavaan* package. Spatial preprocessing was facilitated by *sf, spdep*, and *rgeos*. The spatial mesh for the SPDE model was constructed using inla.mesh.2d(), while the stacking of covariates, spatial, and temporal structures was done using *inla.stack*(). The INLA framework was chosen over traditional MCMC methods due to its computational efficiency and accurate approximation of marginal posteriors, particularly in complex latent Gaussian models (18).

## 3 Empirical results

### 3.1 Descriptive statistics

The distribution of the syndemic scores, derived from the latent variable model incorporating HIV and other STI indicators, revealed moderate variability across individuals. Scores ranged from −0.481 to 0.630, with a mean of −0.009 (SD = 0.248), a median of −0.060, and an interquartile range of 0.270. Most participants were clustered near the average, while a smaller subset experienced considerably higher co-occurring burdens. Negative values represent below-average syndemic burden, whereas positive values indicate above-average burden, with larger absolute values reflecting greater deviation from the population mean. The distribution was approximately symmetric with slight negative skew (skewness = −0.388, kurtosis = −0.243), and visual inspection of histograms and density plots confirmed that it was sufficiently close to normal to justify reporting means and standard deviations.

Table 1 presents a descriptive summary of the latent syndemic score stratified by key socio-demographic, behavioral, and structural covariates among 20,048 individuals residing in Vulindlela and Greater Edendale areas of uMgungundlovu Municipality. The mean syndemic scores varied considerably across age groups, gender, education levels, and behavioral characteristics.

**Table 1:** Descriptive summary statistics of syndemic score of HIV and other STIs by each covariate among individuals in Vulindela and Greater Edendale areas in uMgungundlovu municipality.

**Covariate**	**Count = 20,048**	**Mean**	**SD**
**Age group**			
15–19	3,567	−0.168	0.228
20–24	4,236	−0.047	0.222
25–29	3,494	0.028	0.239
30–34	2,806	0.080	0.230
35–39	2,273	0.077	0.234
40–44	1,931	0.067	0.237
45–49	1,741	−0.005	0.251
**Gender**			
Female	12,606	0.030	0.238
Male	7,442	−0.075	0.252
**Highest education**			
Complete secondary	7,988	−0.001	0.233
Incomplete secondary	9,153	−0.009	0.260
No schooling/creche/pre-primary	521	−0.165	0.282
Primary (grade 1–7)	1,157	0.007	0.263
Tertiary (diploma/degree)	1,229	−0.010	0.208
**Main income**			
No income	1,375	−0.091	0.288
Other non-farming income	1,296	0.008	0.239
Pension or grants	5,892	0.022	0.239
Remittance	300	0.001	0.241
Salary and/or wage	11,185	−0.017	0.246
**Marital status**			
Married	2,960	0.056	0.214
Single	17,088	−0.020	0.252
**Forced first sex**			
No	19,472	−0.011	0.248
Yes	576	0.045	0.258
**Sex ever**			
No	2,846	−0.230	0.224
Yes	17,202	0.027	0.233
**Away from home**			
No	18,167	−0.009	0.249
Yes	1,881	−0.008	0.246
**Number of sexual partners**			
1	15,487	−0.021	0.250
2	2,441	0.021	0.229
3+	2,120	0.042	0.246
**Alcohol consumption**			
No	15,078	−0.014	0.246
Yes	4,970	0.005	0.255
**Meal cuts**			
No	14,073	−0.026	0.252
Yes	5,975	0.032	0.235
**Length in community**			
Always	13,747	−0.033	0.253
Less than 1 year	623	0.029	0.242
More than 1 year	5,678	0.043	0.229
**Meal skip**			
No	16,692	−0.017	0.250
Yes	3,356	0.030	0.238
**Run out of money**			
No	12,964	−0.027	0.253
Yes	7,084	0.023	0.237
**Depression treatment**			
No	19,439	−0.011	0.249
Yes	609	0.036	0.231
**Accessed health care**			
No	9,507	−0.042	0.251
Yes	10,541	0.021	0.242

Higher mean syndemic scores were observed among individuals aged 30–39 years, those with primary education, and individuals who reported sexual activity, multiple sexual partners, or experiences of food insecurity (meal skipping or cutting meals). Females, married individuals, and those who had accessed healthcare services or received treatment for depression also exhibited higher mean scores, potentially reflecting greater detection or underlying vulnerability.

Conversely, adolescents (15–19 years), those with no sexual history, and individuals with no schooling or no reported income showed the lowest mean syndemic scores. Notably, participants reporting three or more sexual partners and those experiencing food insecurity had elevated scores, suggesting a compounding burden from both behavioral and structural vulnerabilities.

These descriptive patterns provide initial insights into the distribution of syndemic burden and inform the selection of covariates for further multivariable spatial and spatio-temporal modeling.

### 3.2 Spatial clustering of syndemic burden

To examine the spatial clustering of the syndemic burden across enumeration areas (EAs), we employed two complementary local spatial statistics: Local Moran's *I* (LISA) and Getis-Ord Gi^*^. These methods identify statistically significant local patterns of spatial association, revealing areas with unusually high (hotspots) or low (coldspots) syndemic scores relative to their neighbors.

**Figure 1:**
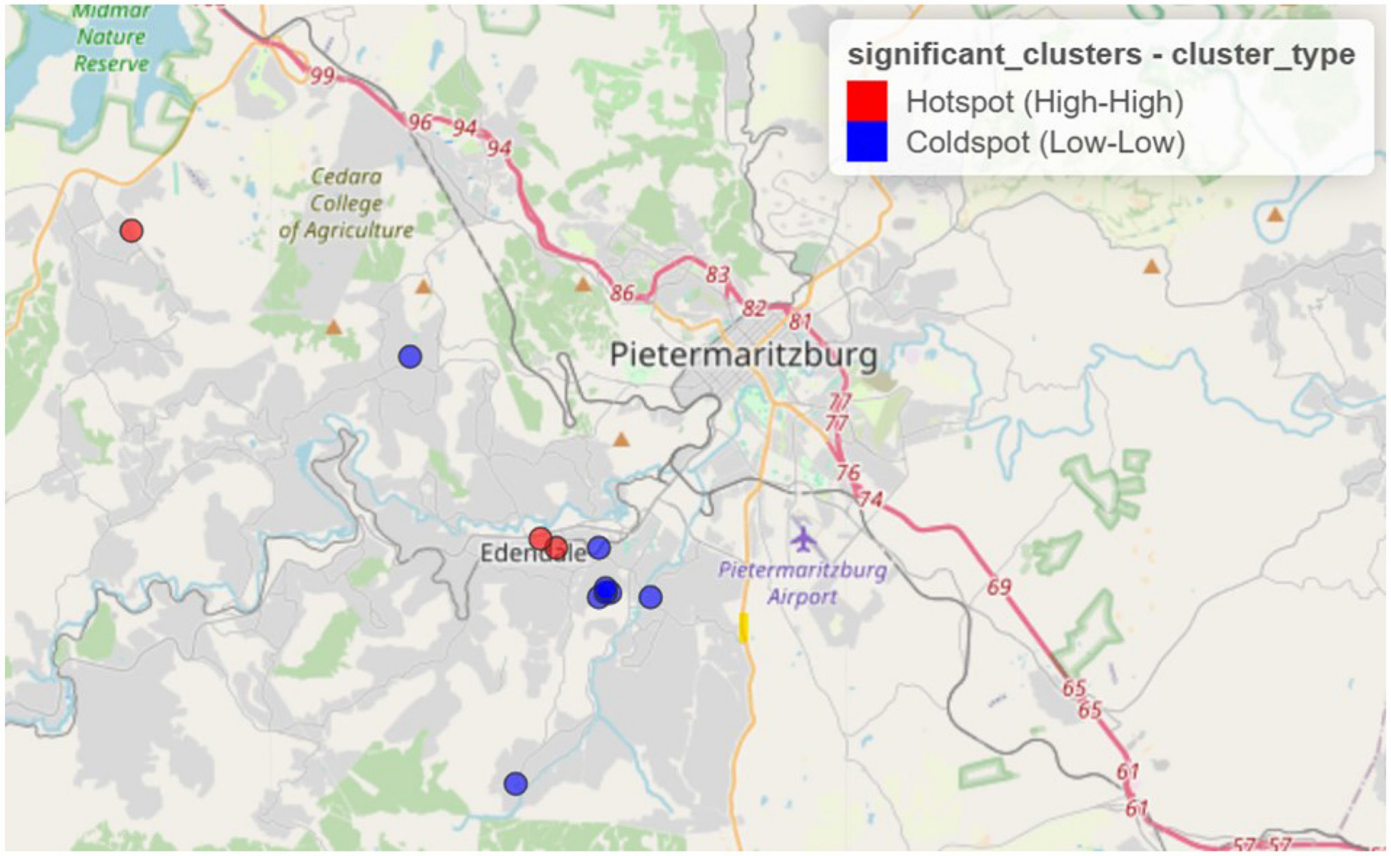
Spatial clustering of syndemic burden of HIV and other STIs in Vulindela and Greater Edendale area based on local Moran's *I* (https://drive.google.com/file/d/1rziEUI2RP2-p71QZiWzi794RQuKRlbsf/view?usp=sharing).

Figure 1 displays the spatial distribution of statistically significant clusters based on Local Moran's *I* (LISA) method. The results from LISA identified 11 significant clusters, including three high–high clusters (hotspots) and eight low–low clusters (coldspots).

**Figure 2:**
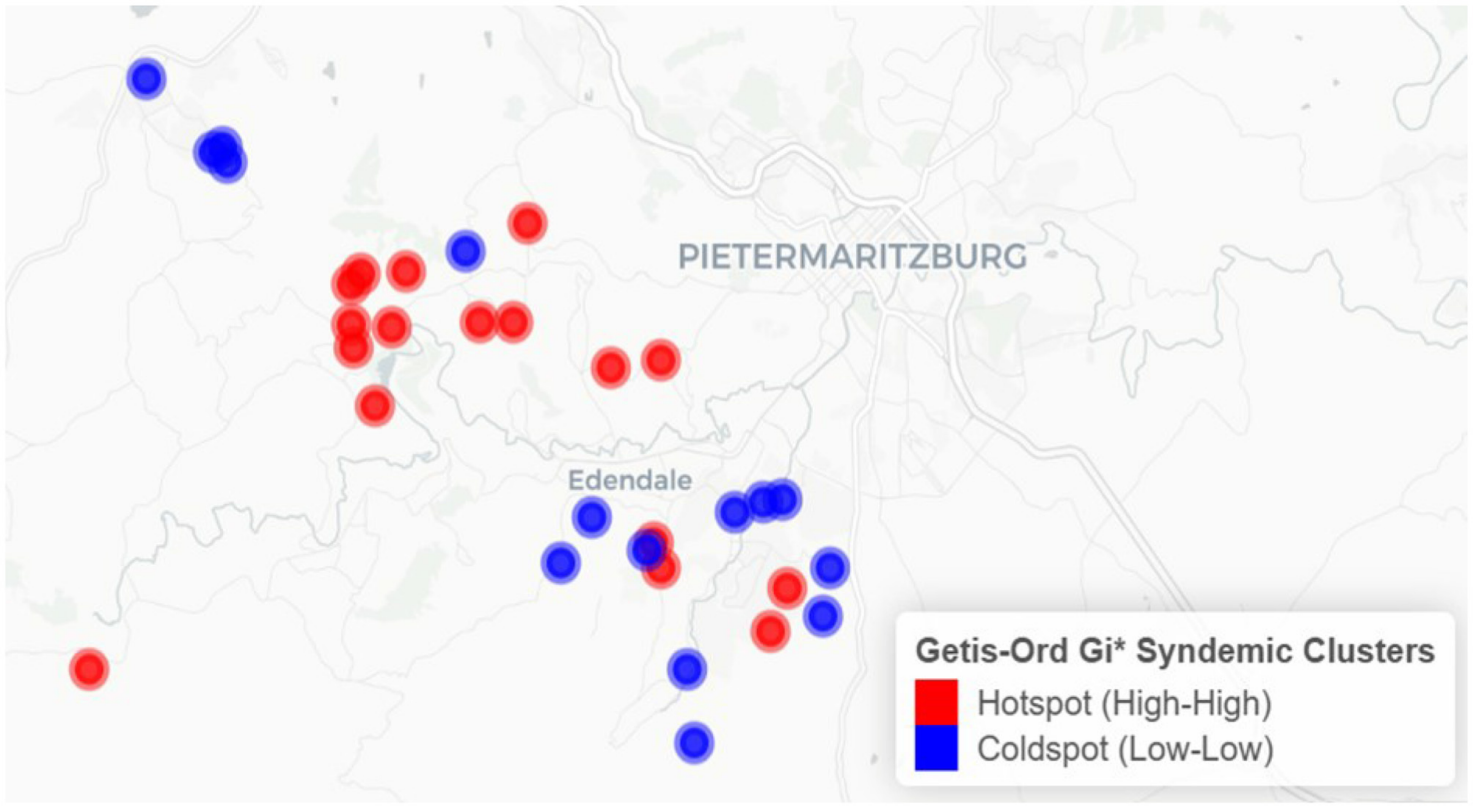
Spatial clustering of syndemic burden of HIV and other STIs in Vulindela and Greater Edendale area based on Getis-Ord Gi* (https://drive.google.com/file/d/1UKp0sxkCW4TLc9BOSalkOM2S1DjsLyoK/view?usp=sharing).

The results from the Getis-Ord Gi^*^ analysis are displayed in Figure 2. Compared to LISA findings, the Getis-Ord Gi^*^ statistic detected 32 significant clusters, comprising 17 hotspots and 15 coldspots.

Clusters identified by both methods indicate that areas with elevated syndemic burden are surrounded by similarly high-burden areas, while low-burden clusters reflect spatially contiguous areas of lower syndemic scores.

Hotspots were predominantly concentrated in and around Edendale, indicating a localized area of entrenched syndemic vulnerability. Coldspots, in contrast, were more frequently observed in peripheral and outlying areas to the northwest, west, and southwest of the study region, with some also adjacent to Edendale. These patterns highlight the geographically clustered nature of syndemic burden, suggesting priority zones for targeted public health interventions.

Table 2 presents a summary of the number of significant clusters identified per year using both LISA and Getis-Ord Gi^*^ statistics.

**Table 2:** Summary table of the number of significant clusters per year for both Local Moran's *I* (LISA) and Getis-Ord Gi^*^ analyses.

**Year**	**LISA hotspots**	**LISA coldspots**	**Total (LISA)**	Getis-Ord Gi^*^hotspots	Getis-Ord Gi^*^coldspots	Total (Getis-Ord Gi^*^)
2014	1	7	8	10	11	21
2015	2	1	3	7	4	11
**Total**	**3**	**8**	**11**	**17**	**15**	**32**

The variation in the number of clusters detected by LISA and Gi^*^ highlights their complementary perspectives: LISA captures local spatial autocorrelation, while Gi^*^ emphasizes the intensity of clustering. Using both approaches strengthens confidence in the observed spatial patterns.

### 3.3 Spatio-temporal interaction effects

The temporal component of the model, fitted with a RW1 prior, revealed meaningful year-to-year differences in syndemic burden. Relative to the overall mean effect, the posterior mean for 2014 was slightly negative (mean = −0.016, 95% CrI: −0.021 to −0.011), while 2015 showed a positive deviation (mean = 0.016, 95% CrI: 0.011 to 0.021). This pattern indicates a modest but credible increase in the underlying syndemic burden between 2014 and 2015. The temporal effect was estimated independently of the spatio-temporal interaction, reinforcing that both spatial clustering and year-to-year changes contributed to the observed heterogeneity.

**Figure 3:**
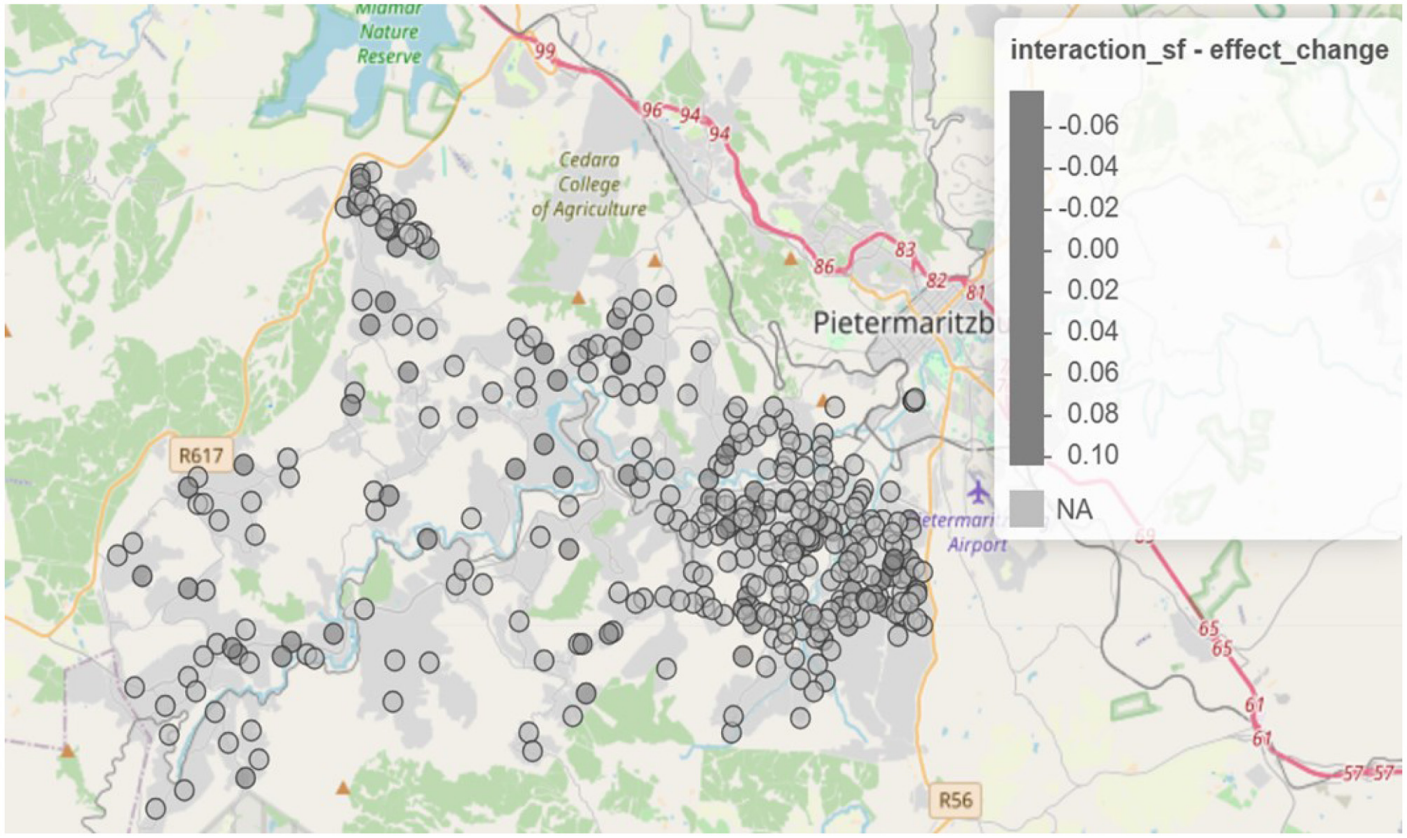
Spatio-temporal change in interaction effects from 2014 to 2015 (https://drive.google.com/file/d/1oB0sedSdUEkvutXqsVx5j5upX1q05ZGq/view?usp=sharing).

The spatio-temporal interaction component of the Bayesian hierarchical model revealed significant localized changes in the syndemic burden between 2014 and 2015. As shown in Figure 3, areas with increasing interaction effects were primarily concentrated in the eastern and central parts of the study region. These areas exhibited growing syndemic clustering over time, potentially signaling intensifying co-epidemics of HIV and STIs. Conversely, there are regions where interaction effects decreased, suggesting a possible attenuation of syndemic burden or improved health interventions. Areas with declining interaction effects were observed mainly in parts of the northwestern and southwestern regions.

**Figure 4:**
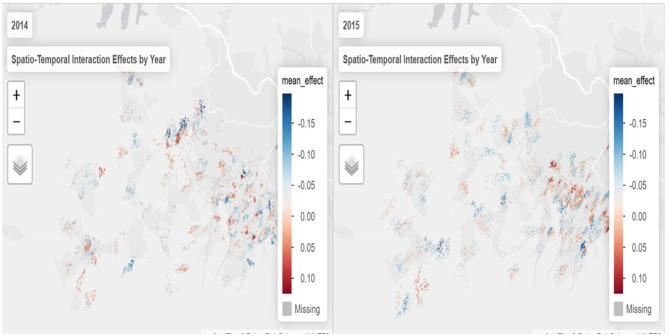
Spatio-temporal interaction effects by year (https://drive.google.com/file/d/1gAyM1jD0Buc8SZMO_nyHC9KOAFJaTNC6/view?usp=sharing).

To further explore annual dynamics, Figure 4 presents maps of spatio-temporal interaction effects separately for 2014 and 2015. In 2014, hotspots of syndemic interactions were more scattered, with pronounced clustering in the northwest. By 2015, these clusters shifted and intensified, particularly in peri-urban areas, as indicated by the darker red zones. Meanwhile, regions that transitioned to blue shades reflect localized reductions in syndemic interaction effects.

Together, these spatial patterns suggest that the syndemic burden is not static but evolves unevenly across both space and time, underlining the importance of targeted, place-based interventions. The shifting clusters also point to underlying social and structural drivers that may have intensified or diminished over the study period.

Temporal dynamics further support this interpretation. In 2014, coldspots were more prominent, while 2015 exhibited an increase in the number and intensity of hotspots, indicating a temporal shift in the spatial distribution of syndemic burden. This year-to-year variation underscores the need to account for temporal change when identifying priority areas for public health interventions. The observed spatio-temporal heterogeneity also reinforces the value of including space–time interaction terms in the Bayesian model to capture evolving geographic vulnerability.

### 3.4 Syndemic model results

Table 3 presents the posterior estimates and corresponding 95% credible intervals for covariates associated with syndemic burden, derived from the Bayesian spatio-temporal model. These results reflect the relative contribution of individual- and household-level factors after accounting for spatial structure, temporal trends, and space-time interactions. Covariates with credible intervals that do not cross zero are considered statistically significant contributors to variation in the latent syndemic score.

**Table 3:** Posterior estimates and 95% credible intervals for covariates associated with syndemic burden of HIV and other STIs from the Bayesian spatio-temporal model.

**Covariate**	**Estimate (β)**	**95% CI lower**	**95% CI upper**
Intercept	**−0.167**	−0.179	−0.156
**Age group (ref: 15–19)**			
20–24	**0.059**	0.048	0.069
25–29	**0.117**	0.106	0.129
30–34	**0.161**	0.149	0.173
35–39	**0.166**	0.153	0.179
40–44	**0.155**	0.141	0.168
45–49	**0.094**	0.080	0.109
**Gender (ref: female)**			
Male	**−0.094**	−0.101	−0.088
**Highest education (ref: complete secondary)**			
Incomplete secondary	**0.013**	0.006	0.019
No schooling/creche/pre-primary	**−0.091**	−0.111	−0.071
Primary (grade 1–7)	−0.005	−0.018	0.009
Tertiary (diploma/degree)	−0.010	−0.022	0.003
**Main income (ref: no income)**			
Other non-farming income	**0.064**	0.047	0.080
Pension or grants	**0.065**	0.052	0.078
Remittance	**0.068**	0.041	0.095
Salary and/or wage	**0.041**	0.029	0.053
**Marital status (ref: married)**			
Single	**0.011**	0.002	0.021
**Forced first sex (ref: no)**			
Yes	**0.037**	0.019	0.054
**Sex ever (ref: no)**			
Yes	**0.152**	0.142	0.163
**Away from home (ref: no)**			
Yes	0.003	−0.007	0.014
**Number of sexual partners (ref: 1)**			
2+	**0.028**	0.023	0.033
**Alcohol consumption (ref: no)**			
Yes	**0.012**	0.004	0.019
**Meal cuts (ref: no)**			
Yes	**0.013**	0.003	0.023
**Length in community (ref: always)**			
Less than 1 year	0.026	0.009	0.044
More than 1 year	0.017	0.009	0.024
**Meal skip (ref: no)**			
Yes	0.009	−0.001	0.019
**Run out of money (ref: no)**			
Yes	0.002	−0.007	0.011
**Depression treatment (ref: no)**			
Yes	0.031	0.013	0.049
**Accessed health care (ref: no)**			
Yes	**0.031**	0.025	0.038

Bold values indicate significant posterior effects.

Several sociodemographic and behavioral factors were significantly associated with the syndemic score. Compared to individuals aged 15–19 years, those aged 20–24 (β = 0.059; 95% CI: 0.048, 0.069), 25–29 (β = 0.117; 95% CI: 0.106, 0.129), 30–34 (β = 0.161; 95% CI: 0.149, 0.173), 35–39 (β = 0.166; 95% CI: 0.153, 0.179), 40–44 (β = 0.155; 95% CI: 0.141, 0.168), and 45–49 (β = 0.094; 95% CI: 0.080, 0.109) showed significantly higher syndemic burden, indicating increasing vulnerability with age. Males exhibited lower syndemic scores than females (β = −0.094; 95% CI: −0.101, −0.088).

Education level was associated with syndemic burden. Participants with incomplete secondary education had significantly higher scores (β = 0.013; 95% CI: 0.006, 0.019) compared to those who completed secondary schooling, whereas those with no schooling or only pre-primary education had lower scores (β = −0.091; 95% CI: −0.111, −0.071), possibly reflecting underdiagnosis or limited access to health services. Regarding income, reliance on remittances (β = 0.068; 95% CI: 0.041, 0.095), pensions or grants (β = 0.065; 95% CI: 0.052, 0.078), other informal income (β = 0.064; 95% CI: 0.047, 0.080), or salary and/or wage (β = 0.041; 95% CI: 0.029, 0.095) was associated with elevated syndemic scores relative to those reporting no income.

Beyond socioeconomic factors, behavioral and psychosocial indicators also showed strong associations. Having ever had sex (β = 0.152; 95% CI: 0.142, 0.163) and a higher number of sexual partners (β = 0.028; 95% CI: 0.023, 0.033) were linked to greater syndemic burden. Experiencing forced sex at sexual debut (β = 0.037; 95% CI: 0.019, 0.054) and shorter duration of residence in the community (β = 0.026; 95% CI: 0.009, 0.044 for < 1 year) were additional risk factors. Access to healthcare (β = 0.031; 95% CI: 0.025, 0.038) and receiving treatment for depression (β = 0.031; 95% CI: 0.013, 0.049) were also positively associated with syndemic scores.

Caution is warranted in interpreting some predictors. Although access to healthcare and depression treatment were significantly associated with higher syndemic scores, these relationships do not necessarily imply causation. Rather, they may reflect increased engagement with the health system or prior diagnosis, serving as proxies for individuals already experiencing syndemic conditions. Distinguishing true predictors from proxies or outcomes is essential in syndemic analyses to avoid misleading conclusions.

### 3.5 Model diagnostics and performance

The following section presents diagnostic evaluations to confirm the credibility of the Bayesian spatio-temporal model. To assess model adequacy and convergence, several diagnostic metrics were evaluated.

#### 3.5.1 Model selection and fit

Table 4 summarizes the model fit statistics for the two candidate models. Model 2, which incorporated explicit penalized complexity (PC) priors, yielded marginally lower DIC and WAIC values compared to Model 1, indicating slightly better fit. Given that the differences were modest, this comparison provides reassurance that the findings are not sensitive to prior specification.

**Table 4:** Model selection criteria summary for the two competing models.

**Model**	**DIC**	**WAIC**
1	−4,976.66	−4,973.83
2	−4,987.63	−4,983.35

The substantive results presented in the paper are therefore based on Model 2, which we selected as the model of best fit. Importantly, posterior estimates of fixed effects, temporal dynamics, and spatial patterns were highly consistent across both models, indicating that the substantive conclusions are robust to prior and hyperparameter specification.

#### 3.5.2 Predictive accuracy

**Figure 5:**
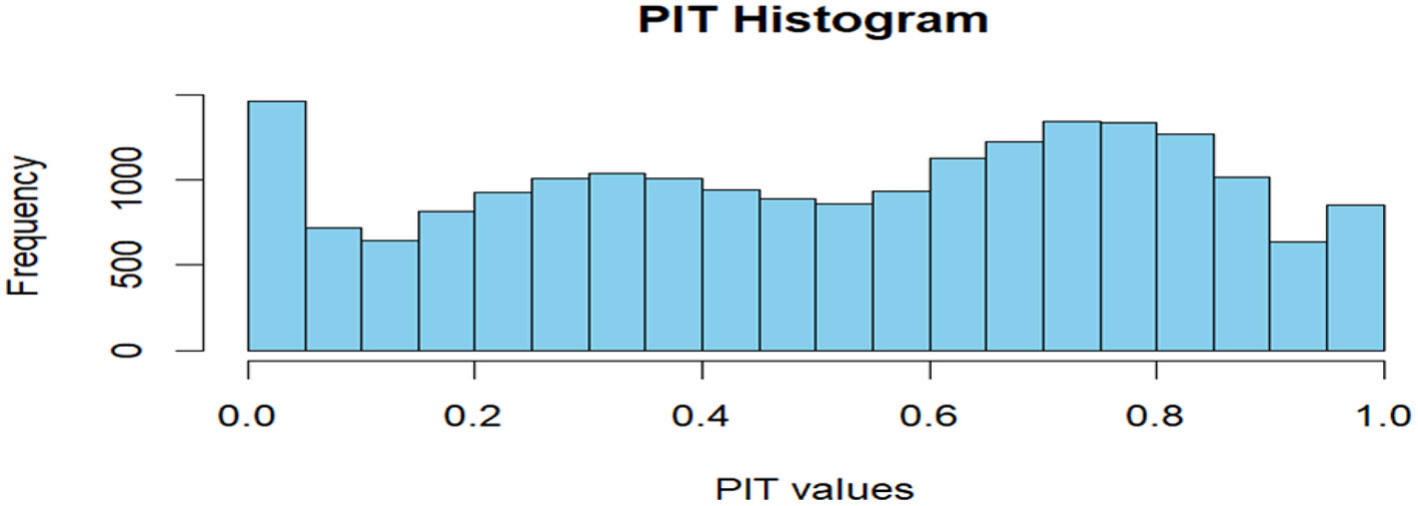
PIT histogram.

CPO and PIT values were computed to assess model predictive performance and calibration. Figure 5 shows the PIT histogram.

**Figure 6:**
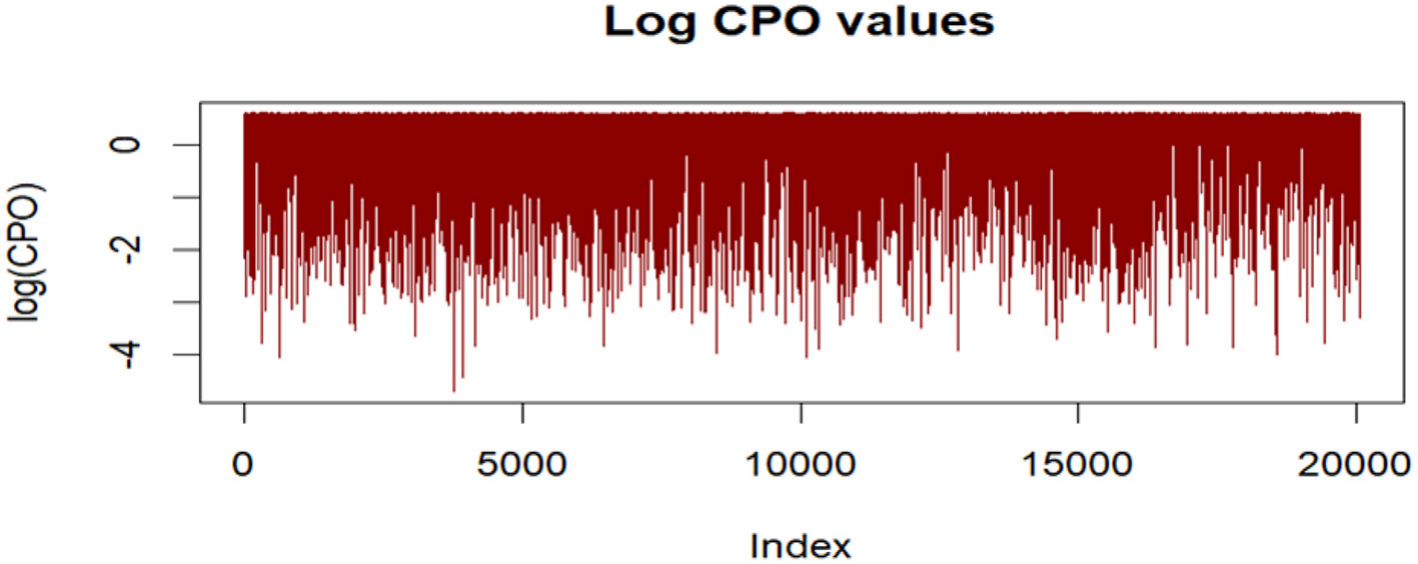
Log CPO values plot.

The histogram of PIT values approximated a uniform distribution, suggesting good model calibration. Additionally, no extreme outliers were identified in log(CPO) plots (Figure 6), supporting satisfactory predictive adequacy.

#### 3.5.3 Convergence diagnostics

**Figure 7:**
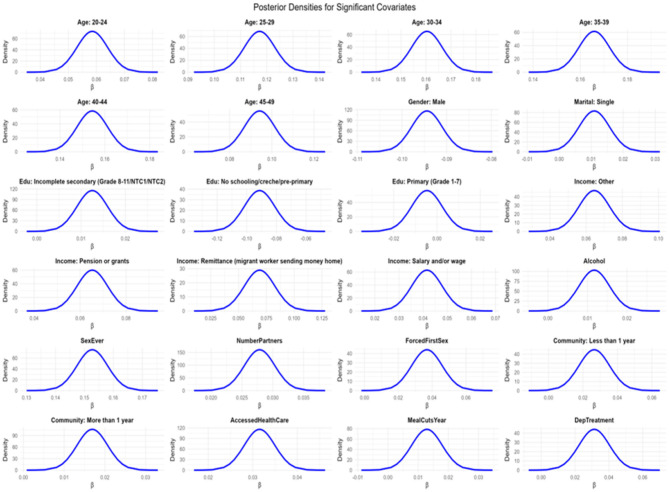
Density plots for significant syndemic score covariates for HIV and other STIs.

The fixed effects exhibited excellent convergence, as assessed by the absolute differences between posterior means and modes, all of which were less than 5 × 10^−3^. The posterior density plots were sharply peaked and unimodal (Figure 7), further supporting stability of estimation. Moreover, the Kullback–Leibler divergence (KLD) values for all fixed effects were zero, indicating that the posterior marginals were very well approximated by Gaussian distributions. Together, these diagnostics suggest robust and reliable parameter estimation.

Figure 7 shows the density plots for the Bayesian spatio-temporal model, used to assess parameter convergence and distribution of posterior samples.

The density plots show smooth, unimodal distributions, further confirming stable posterior estimation.

#### 3.5.4 Hyperparameter assessment

The model's hyperparameters indicated that the residual variance was modest (σ^2^ ≈ 0.044), supporting good overall model fit. Temporal variability was small (σ^2^ ≈ 0.0019), suggesting that year-to-year trends were relatively smooth and largely captured by the fixed effects. The estimated spatial range was broad (median ≈ 1.686 km; 95% CrI: 0.022–261 km) but highly uncertain, implying weak, though possible, evidence of structured spatial autocorrelation. The posterior standard deviation for the spatial field was small (≈ 0.035; 95% CrI: 0.011–0.077), indicating limited but non-negligible residual clustering. Space–time random effects had a comparable variance (σ^2^ ≈ 0.0014), suggesting that localized spatio-temporal interactions contributed meaningfully to unexplained variability. Overall, these results indicate that while residual spatial and temporal effects were modest, space–time interactions played a notable role in shaping the observed syndemic patterns.

## 4 Discussion

This study advances our understanding of syndemic interactions involving HIV and other STIs in KwaZulu-Natal by applying a Bayesian spatio-temporal structural equation model to repeated cross-sectional data. By constructing a latent syndemic variable, we were able to capture co-occurring disease burdens and identify distinct demographic, behavioral, psychosocial, and structural predictors of heightened vulnerability. The analysis further revealed substantial spatial and temporal heterogeneity in syndemic patterns, underscoring the importance of geographically and temporally tailored public health strategies.

Age emerged as a key determinant of syndemic burden. Individuals aged 35–39 years had the highest latent scores, consistent with national data showing elevated HIV and TB burdens in this group (3, 10). Gender differences were also notable: female participants exhibited higher syndemic scores than males, aligning with findings from (23) on structural gender disparities, including hegemonic masculinity, male violence, and gendered power inequities. These findings are also consistent with (24), who reported significantly higher STI prevalence among females compared to males in KwaZulu-Natal.

Educational attainment played a critical role. Incomplete secondary education was significantly associated with higher syndemic scores, reinforcing earlier studies that highlighted the protective effect of higher education (25, 26). Interestingly, participants with no formal education showed slightly lower syndemic scores, possibly due to underreporting or limited healthcare access, highlighting the need for improved outreach in underrepresented populations.

Socioeconomic vulnerability also contributed to syndemic burden. Participants relying on remittances or social grants reported higher scores. These income types, while supporting subsistence, may reflect broader structural inequities that facilitate syndemic interactions.

Behavioral and psychosocial risk factors such as alcohol use, early sexual debut, and multiple sexual partnerships were all significantly associated with higher syndemic burden, consistent with previous studies (27, 28).

Mobility indicators showed that individuals who had lived in their communities for less than 1 year experienced significantly greater syndemic burden. This supports findings, that linked migration to disrupted healthcare continuity and heightened disease risk (29–31).

Spatial analyses revealed marked clustering of syndemic burden. Using Local Moran's *I*, 27 significant spatial clusters were detected, comprising 13 hotspots (high–high) and 14 coldspots (low–low). The Getis-Ord Gi^*^ statistic identified 29 clusters, including 17 hotspots and 12 coldspots. The consistency of these patterns between 2014 and 2015 indicates temporal stability in geographic disparities, consistent with findings from previous studies (9, 32). Persistent hotspots were concentrated in and around Edendale, reflecting entrenched syndemic vulnerability. Coldspots were more frequently observed in peripheral areas to the northwest, west, and southwest of the study region, with some also adjacent to Edendale.

Spatio-temporal modeling further revealed that syndemic clustering extended over distances of up to 1.686 km, suggesting regional diffusion of risk likely shaped by social, infrastructural, or healthcare access gradients. This finding is consistent with previous research (33), demonstrating the spatial heterogeneity and diffusion in HIV transmission patterns in KwaZulu-Natal, influenced by mobility and geographic disparities in healthcare access. Temporal dynamics were also evident: coldspots were more numerous in 2014, while 2015 showed a slight increase in LISA hotspots and a decrease in Gi^*^ hotspots, highlighting shifts in the spatial distribution of syndemic burden. These patterns align with observations by (26) and (32), who found that the burden of HIV and related infections is shaped by changing spatial and temporal risk environments. Such shifts reinforce the importance of dynamic, time-sensitive intervention strategies and support the inclusion of space–time interaction terms in the modeling framework, as emphasized in spatial epidemiological studies (16) and (34).

Our findings align with and extend prior spatial epidemiology research in KwaZulu-Natal. Modeling using Bayesian CAR models mapped and showed overlapping HIV and STI clusters in peri-urban Durban (35), while geoadditive INLA models identified predictors of unsuppressed HIV viral loads (36). Our latent syndemic framework moves beyond these approaches by integrating multiple indicators into a composite index, capturing the synergistic nature of overlapping syndemic of HIV and STIs.

The application of Bayesian latent variable models integrated with spatio-temporal analysis provided a robust framework for identifying persistent syndemic of HIV and STIs hotspots and their drivers. The model diagnostics, including DIC (−4,987.63) and WAIC (−4,983.35), supported good fit. The spatial field had an estimated range of approximately 1.686 km, and the temporal component captured year-to-year variation through a first-order random walk. The low variance of space-time interaction effects (σ^2^ ≈ 0.0014) indicated that much of the spatio-temporal variability was already accounted for by the structured components.

Altogether, this study demonstrates the power of combining syndemic theory with modern spatial statistics to uncover actionable insights. Persistent geographic hotspots and their social and behavioral correlates help to inform integrated, multi-sectoral responses. These must go beyond disease-specific silos to address the interlinked drivers of poor health in high-burden settings, such as KwaZulu-Natal, ultimately advancing health equity and the goals of syndemic-informed public health policy. From a public health perspective, these findings highlight the importance of geographically targeted interventions, such as prioritizing HIV testing, treatment, and prevention services in identified hotspots. They also underscore the need to strengthen education, gender equity, and poverty alleviation initiatives as structural interventions that can mitigate overlapping vulnerabilities. By linking spatial epidemiological evidence with broader social policy, the study provides a foundation for more efficient allocation of resources and the design of tailored, community-centered programmes.

### 4.1 Strengths and limitations

In interpreting these findings, it is important to consider both the strengths and limitations of the study. First, missing data presented a challenge. While multiple imputation was applied for variables with moderate levels of missingness, residual bias due to imputation cannot be fully ruled out. The number of indicators available for the latent construct was also limited, reflecting constraints of the survey instrument. Although the chosen indicators capture essential domains, the construct may not fully represent the wider underlying phenomenon. The inclusion of individual survey weights in the INLA model enhances the representativeness of the estimates and improves generalisability to the broader population, although some residual limitations inherent to survey-based data may remain. Further, only a limited number of survey waves were available, which restricted the ability to examine longer-term trends. Nevertheless, the available waves provided valuable insights into temporal patterns within the study period.

Second, as with all Bayesian approaches, the modeling is influenced by prior specification. Although we applied penalized complexity (PC) priors, which are designed to be weakly informative and to guard against overfitting, some degree of subjectivity in prior selection remains inherent to Bayesian inference. At the same time, the Bayesian framework offered important strengths: it allowed explicit incorporation of spatial and temporal dependence, coherent quantification of uncertainty, and estimation of latent constructs that would be difficult to address under frequentist approaches.

Finally, while repeated cross-sectional data enabled robust spatio-temporal inference, causal relationships cannot be definitively established. Future longitudinal studies are needed to confirm the observed associations and dynamics.

Despite these limitations, the study has notable strengths. Integrating syndemic theory with advanced Bayesian spatio-temporal structural equation modeling provided a novel framework for capturing complex disease interactions across space and time. The modeling strategy also enabled the identification of persistent geographic hotspots and their social and behavioral drivers. Together, these insights advance understanding of overlapping epidemics and highlight priority areas for intervention.

## 5 Conclusion

Taken together, despite these limitations, our findings provide important insights into the syndemic dynamics of HIV and other STIs in KwaZulu-Natal through the application of a Bayesian spatio-temporal structural equation modeling framework. By constructing a latent syndemic score from multiple disease indicators and incorporating both spatial and temporal components, we identified persistent high-burden hotspots, and a range of individual and structural-level risk factors associated with syndemic vulnerability.

The analysis revealed consistent spatial clustering of syndemic burden across enumeration areas, with stable hotspots observed over two consecutive years (2014 and 2015). This temporal stability underscores the entrenched nature of overlapping epidemics in specific geographic regions. Key covariates, such as age, gender, education, income sources, sexual behaviors, experiences of forced sex, depression treatment, food insecurity, mobility, and healthcare access, were significantly associated with elevated syndemic burden, reaffirming the interdependent nature of these health and social risks.

Methodologically, the integration of latent variable modeling with spatio-temporal analysis provides a robust and nuanced framework for understanding and visualizing complex disease interrelationships in high-burden settings. The identification of persistent geographical hotspots offers critical insight for the development of spatially targeted, multi-sectoral interventions that address not only clinical outcomes but also the social and structural determinants that sustain syndemics.

These findings have substantial public health implications. The presence of stable syndemic hotspots highlights the need for geographically prioritized interventions in KwaZulu-Natal. The associations with structural vulnerabilities, such as food insecurity, mobility, and mental health, point to the necessity of comprehensive strategies that extend beyond biomedical care. Additionally, the elevated burden among individuals aged 20–39 underscores the urgency of youth-focused and gender-sensitive prevention initiatives.

Future research should prioritize longitudinal tracking to clarify causal mechanisms and evaluate the impact of integrated health and social interventions in hotspot regions. As health systems in resource-limited settings increasingly face the complexity of syndemic interactions, the analytical approach presented here offers a scalable tool for precision public health planning and policy.

## Data Availability

The raw data supporting the conclusions of this article will be made available by the authors, without undue reservation.
